# A Novel Tubeless Urinary Catheter Protocol Enhanced Recovery After Minimally Invasive Lung Surgery

**DOI:** 10.3389/fsurg.2020.584578

**Published:** 2020-11-09

**Authors:** Weidong Wang, Pinghui Xia, Liang Pan, Jinming Xu, Wang Lv, Jian Hu

**Affiliations:** Department of Thoracic Surgery, The First Affiliated Hospital, School of Medicine, Zhejiang University, Hangzhou, China

**Keywords:** tubeless, urinary catheter, enhanced recovery after surgery (ERAS), urinary tract infection (UTI), lung surgery

## Abstract

**Objectives:** Although previous studies have shown the feasibility of non-intubated techniques, it is unknown whether avoiding urinary catheters can enhance recovery. This study aimed to determine whether the tubeless urinary catheter protocol is feasible and beneficial for minimally invasive lung surgery.

**Methods:** Patients were randomized to the control group, completely tubeless group, and partially tubeless group. A propensity score–matched (PSM) analysis was performed to balance the non-random baseline characteristics. Complications and postoperative recovery were compared. Regression analysis was performed to identify the independent predictors of complications. A nomogram for predicting the risk of non-automatic micturition was constructed and internally validated.

**Results:** One hundred fifty-nine patients were enrolled. The incidence rates of urinary irritation and urinary tract infection (UTI) were significantly lower in the tubeless groups (74.4 vs. 39.5%, *p* < 0.001; 28.2 vs. 8.6%, *p* = 0.001, respectively). The tubeless group had a higher proportion of 0-degree discomfort (81.5 vs. 30.8%, *p* = 0.001) and shorter duration of postoperative hospital stay than the control group (4.59 vs. 5.53 days, *p* < 0.001). No difference was observed in terms of urination retention and urinary incontinence between the tubeless group and the control group. After PSM, the advantages of the tubeless group still existed, and comparing to the partially tubeless group, the completely tubeless group was of even less UTI and more 0-degree discomfort (18.5 vs. 0.0%, *p* = 0.019; 96.3 vs. 59.3%, *p* = 0.002). The tubeless protocol was the only independent protective factor of urinary complications. A nomogram was constructed and showed good predictive ability.

**Conclusions:** The tubeless catheterization protocol led to fewer complications, better compliance, and shorter hospital length of stay. The advantages were more significant with the completely tubeless protocol. The utility of our nomogram can assist clinicians in avoiding risks in performing the tubeless protocol.

## Introduction

Enhanced Recovery After Surgery (ERAS) is an evidence-based multidisciplinary perioperative care and surgical quality improvement that has been shown to minimize the invasiveness of surgery, promote recovery, and reduce complications (1). The concept of ERAS was initially introduced in 2011 and had been developed to accelerate recovery, but most available evidence regarding the benefits of ERAS mainly focuses on gastrointestinal surgery (2).

The use of ERAS in lung resection was introduced 10 years ago and showed favorable results (3). Since the recommendation of including video-assisted thoracoscopic surgery (VATS) in the management of small lung lesions (4), the technique has been significantly improved, resulting in shorter surgical duration and fewer complications (5). Meanwhile, anesthetists and thoracic surgeons are continuing to reduce the invasiveness, which led to the introduction of non-intubated VATS.

Non-intubated VATS is a technique that avoids tube placement after VATS under non-intubated spontaneous ventilation. Although intravenous anesthesia without intubation and early removal of a chest tube were proven to be feasible and advantageous over tube-requiring procedures (6, 7), the optimal protocol for urinary catheterization still remains controversial. While urinary catheterization can be helpful for monitoring urine output and preventing postoperative urinary retention (POUR), it can also reduce patients' comfort and delay postoperative mobilization (8). Besides, about 0.3% of patients suffered iatrogenic injuries of the urethra during the insertion process (9). Furthermore, the risk of catheter-associated urinary tract infection (UTI) increases with prolonged duration of the indwelling urinary catheter (10). Although the guidelines of the ERAS Society state that a urinary catheter is unnecessary if its sole purpose is to monitor urine output, relevant evidence is unconvincing, and a recommendation on the timing of catheter removal or the criteria for patients who do not need urinary catheterization cannot be made (11).

As urinary catheter management is an important component of ERAS, it is necessary to develop an optimal protocol for patients who do not need urinary catheterization. Thus, we conducted a randomized controlled trial to analyze the feasibility of the tubeless urinary catheter protocol, compared it with traditional catheterization for selected patients after VATS lung resection, and evaluated the role of this technique in improving patient recovery.

## Patients and Methods

### Patient Selection and Grouping

This study was registered on the China Clinical Trial Registry Center (ChiCTR-INR-17010816). It was approved by the Medical Ethics Committee of the First Affiliated Hospital, School of Medicine, Zhejiang University; the reference number is 2016-249. Every individual participant had signed an informed consent form for participating in the study.

**Figure 1:**
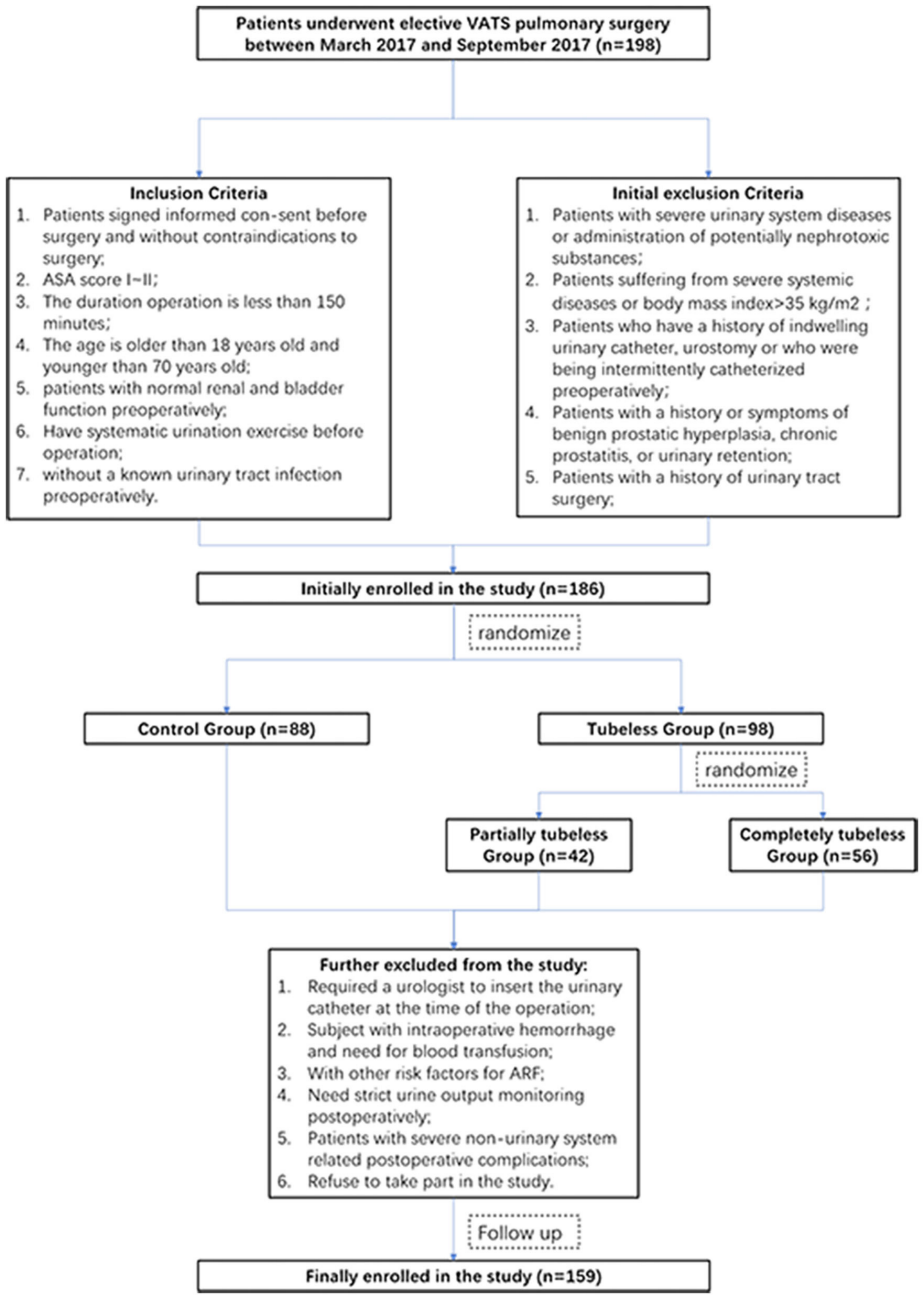
Flowchart of the study.

This study initially included 198 consecutive patients who received minimally invasive lung surgery between March 2017 and September 2017. Upon admission to the hospital, the patients were randomized into two groups (the control group and the tubeless group) according to a random number automatically produced by a computer program. The tubeless group was further randomized into two subgroups (the partially tubeless group and the completely tubeless group) (Figure 1). The eligibility and exclusion criteria are shown in Supplementary Table 1.

A propensity score–matched (PSM) comparative analysis was performed to balance the non-random baseline characteristics among the groups and to decrease the influence of operation and anesthesia on the postoperative recovery outcomes. We adjusted for potential differences between the control group and the tubeless group with 1:1 matching. The same adjustment was also performed in the tubeless group internally. We produced a propensity score for the matched groups using logistic regression based on the patient's age, gender, surgical type, duration of operation, duration of anesthesia, and tumor size with a caliper setting of 0.05.

### Catheterization Procedure

Catheterization was performed by the circulating nurse and surgical team after the induction of anesthesia. The control group had the catheter inserted after the patient was anesthetized and had the catheter removed until postoperative day 2. The partially tubeless group had the catheter inserted after the patient was anesthetized and had the catheter removed as soon as the operation finished. The completely tubeless group did not have a catheter inserted during the perioperative period.

### Anesthesia Method

Anesthesia was induced by propofol [3–6 μg/mL, target control infusion (TCI)], remifentanil (4–6 ng/mL, TCI), and cisatracurium (0.2 mg/kg) and maintained with propofol (3–5 μg/mL, TCI) and remifentanil (4–6 ng/mL, TCI). Intravenous patient-controlled analgesia (hydromorphone 6 mg and flurbiprofen 200 mg) and paravertebral blockade (PB) of 0.4% ropivacaine 20 mL were used to control postoperative pain. Patients were discharged from the post-anesthesia care unit using the Steward score.

### Parameter Collection and Outcome Definition

Patients were asked to finish an online questionnaire (Supplementary Table 2) 48 h after the operation in the tubeless group and 48 h after the catheter was removed in the control group. Professional staff were available to answer questions if any problems occurred in the understanding of the questionnaire. All the participants were preoperatively informed regarding how to distinguish urinary discomfort from postoperative pain.

The time to get out of bed after surgery is the duration from the end of the operation to the first-time ambulation on the patient's own initiative without help from other people.

POUR is defined as the inability to voluntarily urinate or a residual urine volume >600 mL, diagnosed by bedside ultrasound (Supplementary Figure 1), in patients who felt residual urine and was managed by recatheterization. Patients who were not able to voluntarily urinate but the residual urine volume was <600 mL and can urinate after stimulation such as hot compress therapy or body position changes were defined as induced urination. Both POUR and induced urination were included in non-automatic micturition.

UTI is defined by the presence of symptoms or signs compatible with the standard definition of UTIs without other identified sources of infection along with ≥10^5^ colony-forming units/mL of ≥1 bacterial species in a single urine specimen or in a midstream voided urine specimen from a patient whose urethral catheter had been removed within the previous 48 h. Asymptomatic bacteriuria was also included in the UTI (10).

Pyuria is defined as the presence of ≥10 white blood cells (WBCs) per high-power field (HPF) in the centrifugal urine sample, and hematuria is defined as the presence of ≥3 red blood cells (RBCs) per HPF in the centrifugal urine sample.

Patients who suffered from POUR, UTI, urinary incontinence, or II- to III-degree discomfort were defined as having urinary complications.

The criteria for hospital discharge were as follows: (1) life signs of patients were stable; (2) no significant abnormity was observed in the chest X-ray, blood and urine routine examination, routine biochemistry test, and other necessary tests; (3) patients did not feel significant discomfort by themselves. The duration between the operation and hospital discharge was defined as hospital length of stay (LOS).

### Blind Method

The grouping result was blinded to the medical team and the patients until the beginning of the operation. The patients in the tubeless group were not informed about which subgroup they were allocated in. The data of the questionnaire, the perioperative parameters, and the results of laboratory examinations were collected by an independent medical staff who was blinded to the grouping result.

### Statistical Analysis

The associations of the risk of non-automatic micturition in patients with tubeless catheterization were evaluated by the use of logistic regression analysis. Based on the results from the regression analysis, a nomogram for non-automatic micturition probability was constructed. A calibration comparing the predicted and actual probability of non-automatic micturition was used to validate the predictive accuracy of our nomogram. The nomogram was also subjected to 1,000 bootstrap resamples for internal validation to assess its predictive ability.

Categorical variables were compared using the χ^2^ test, whereas continuous variables were analyzed using the *t* test, Mann-Whitney *U* test, and analysis of variance test. Logistic regression was used to identify risk factors. Two-sided *P* values of <0.05 were considered statistically significant. All analyses were performed using SPSS 22.0 software (IBM, Armonk, NY), GraphPad Prism 5.0 software (GraphPad software, La Jolla, CA), and R 3.6.1 (The R Foundation for Statistical Computing, Vienna, Austria) with the rms statistical package.

## Results

### Study Population

Finally, 159 patients were enrolled. No patient suffered from acute renal dysfunction. There were 78 patients in the control group, 30 patients in the partially tubeless group, and 51 patients in the completely tubeless group. Except for the age and the proportion of females in the control group being higher, there was no significant difference in the baseline characteristics among the groups (Table 1).

**Table 1:** Baseline characteristics of the study population.

**Characteristic**	**Full cohort (*n* = 159)**			**Tubeless group (*n* = 81)**		
	**Control group (*n* = 78)**	**Tubeless group (*n* = 81)**	***P***	**Partially tubeless group (*n* = 30)**	**Completely tubeless Group (*n* = 51)**	***P***
**Age, year, mean ± SD**			*<0.001*			*0.113*
	55.32 ± 8.44	49.96 ± 9.89		52.43 ± 11.61	48.51 ± 8.51	
**Gender, *n* (%)**			*0.023*			0.804
Male	36 (46.2)	23 (28.4)		9 (30.0)	14 (27.5)	
Female	42 (53.8)	58 (71.6)		21 (70.0)	37 (72.5)	
**Side of operation, *n* (%)**			*0.596*			0.782
Left	36 (46.2)	34 (42.0)		12 (40.0)	22 (43.1)	
Right	42 (53.8)	47 (58.0)		18 (60.0)	29 (56.9)	
**Diabetes, *n* (%)**			*1.000*			0.525
Yes	2 (2.6)	2 (2.5)		0 (0.0)	2 (3.9)	
No	76 (97.4)	79 (97.5)		30 (100.0)	49 (96.1)	
**Hypertension, *n* (%)**			*1.000*			0.394
Yes	14 (17.9)	15 (18.5)		7 (23.3)	8 (15.7)	
No	64 (82.1)	66 (81.5)		23 (76.7)	43 (84.3)	
**Surgical type, *n* (%)**			*0.515*			0.247
Lobectomy	39 (50.0)	39 (48.1)		14 (46.7)	25 (49.0)	
Segmentectomy	7 (9.0)	4 (4.9)		0 (0.0)	4 (7.8)	
Wedge resection	32 (41.0)	38 (47.0)		16 (53.3)	22 (43.2)	
**Intraoperative blood loss (mL), mean ± SD**			*0.881*			0.285
	67.27 ± 29.99	66.44 ± 21.54		69.53 ± 21.89	64.63 ± 21.34	
**Pathological diagnosis, *n* (%)**			*0.425*			0.418
Malignance	65 (83.3)	63 (77.8)		25 (83.3)	38 (74.5)	
Benign disease	13 (16.7)	18 (22.2)		5 (16.7)	13 (25.5)	
**Diameter of tumor (cm), mean ± SD**			*0.610*			0.454
	1.50 ± 1.14	1.31 ± 0.84		1.21 ± 0.81	1.39 ± 0.86	
**Lymph node dissection, *n* (%)**			*0.365*			0.307
Yes	61 (78.2)	58 (71.6)		24 (80.0)	34 (66.7)	
No	17 (21.8)	23 (28.4)		6 (20.0)	17 (33.3)	

*SD, standard deviation. The italic “n” indicates number of patients*.

After PSM, 65 pairs of patients were eligible for the comparison between the control group and the tubeless group, and there were 27 pairs of patients enrolled for the comparison within the two tubeless groups. All the baseline characteristics are harmonious between the control group and the tubeless group and between the partially tubeless group and the completely tubeless group (Supplementary Table 3).

### Assessment of Urinary Outcomes

**Figure 2:**
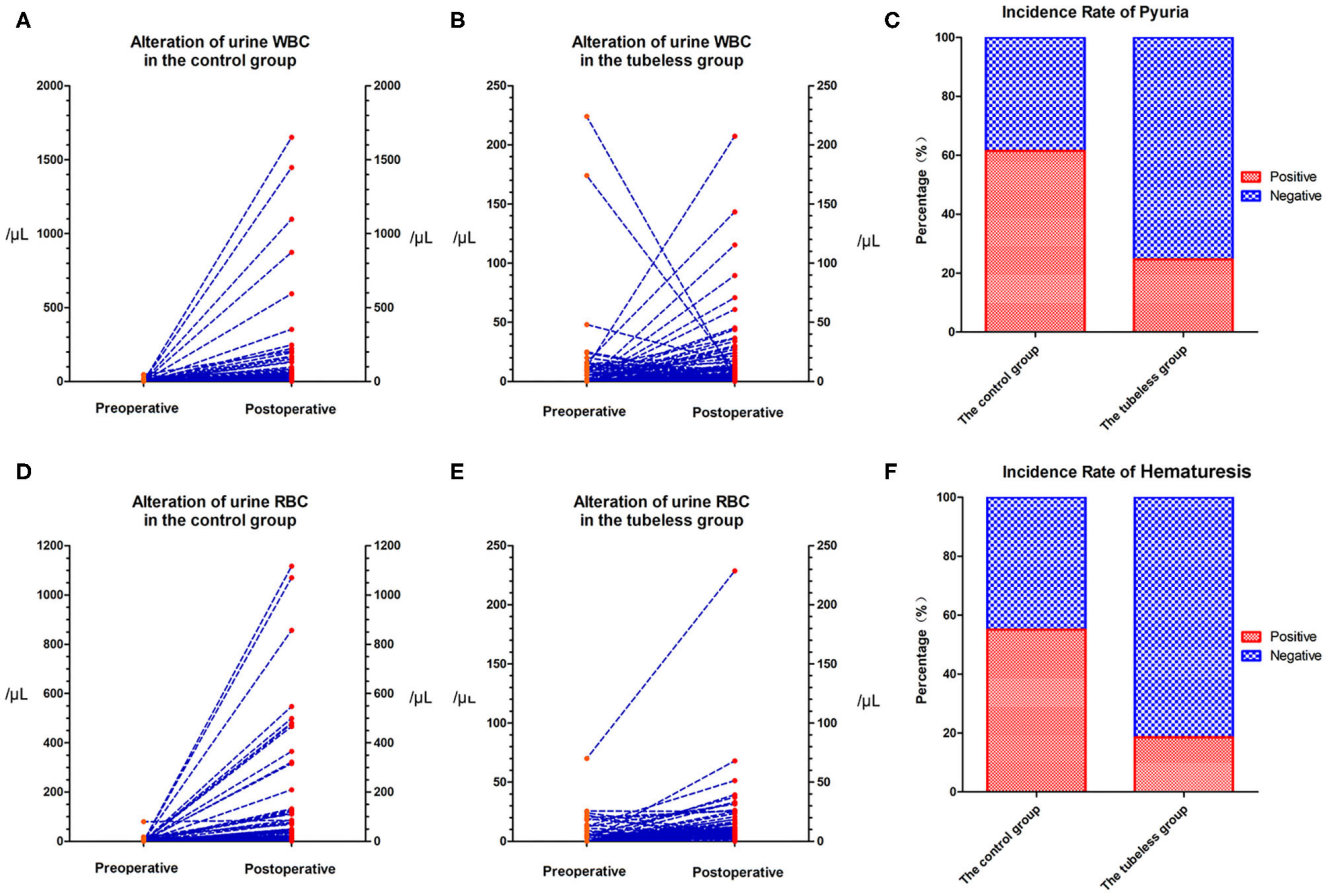
Alteration in urine RBC and WBC levels. **(A)** Alteration of urine WBC level in the control group; **(B)** alteration of urine WBC level in the tubeless group; **(C)** incidence rate of pyuria; **(D)** alteration of urine RBC level in the control group; **(E)** alteration of urine RBC level in the tubeless group; **(F)** incidence rate of hematuresis. RBC, red blood cell; WBC, white blood cell.

The preoperative urine WBC level and urine RBC level were not significantly different between the control group and the tubeless group (*p* = 0.285; *p* = 0.313, respectively). However, the levels in the control group increased sharply after surgery and were significantly higher than those in the tubeless group (*p* < 0.001; *p* < 0.001, respectively) (Supplementary Table 4). The proportion of pyuria and hematuresis were also higher in the control group. But no significant difference was not observed within the two tubeless groups (Figure 2). After PSM, this tendency still existed. Comparing with the control group, the level of postoperative urine WBC and RBC and the incidence rate of pyuria and hematuria were lower in the tubeless group. Additionally, the completely tubeless group was even of lower WBC level than the partially tubeless group (Table 2).

**Table 2:** Urine laboratory examination of our study population after PSM.

**Characteristic**	**Full cohort (*n* = 130)**			**Tubeless group (*n* = 54)**		
	**Control group (*n* = 65)**	**Tubeless group (*n* = 65)**	***P***	**Partially tubeless group (*n* = 27)**	**Completely tubeless group (*n* = 27)**	***P***
**Postoperative urine white blood cell (n/μL), median (min, max)**			<0.001			0.075
	24.8 (0.3, 1,651.0)	6.7 (0.0, 207.4)		8.0 (0.0, 207.4)	3.6 (0.0, 115.5)	
**Postoperative pyuria, *n* (%)**			<0.001			0.735
Positive	40 (61.5)	15 (23.1)		6 (22.2)	5 (18.5)	
Negative	25 (38.5)	50 (76.9)		21 (77.8)	22 (81.5)	
**Postoperative urine red blood cell (n/μL), median (min, max)**			<0.001			0.161
	20.9 (2.0, 1,069.9)	7.9 (0.0, 228.7)		8.8 (0.0, 68.0)	6.7 (0.0, 25.7)	
**Postoperative hematuria, *n* (%)**			<0.001			0.224
Positive	34 (52.3)	10 (15.4)		5 (18.5)	2 (7.4)	
Negative	31 (47.7)	55 (84.6)		22 (81.5)	25 (92.6)	

*Max, maximum; Min, minimum; PSM, propensity score–matched*.

**Figure 3:**
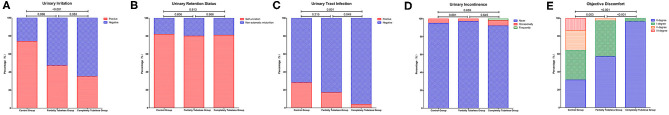
Urinary system–related outcomes among the groups. **(A)** Incidence of urinary irritation; **(B)** Incidence of urinary retention; **(C)** Incidence of urinary tract infection; **(D)** Incidence of uroclepsia; **(E)** Objective discomfort of patients.

Compared to that in the control group, the incidence rates of urinary irritation and UTI were significantly lower in the tubeless group (*p* < 0.001; *p* = 0.001, respectively). The occurrence of UTI in the completely tubeless group was even lower than that in the partially tubeless group (*p* < 0.049). One patient (1/78) in the control group and one patient (1/30) in the partially tubeless group suffered POUR and need for recatheterization. The proportion of patients who had non-automatic micturition or urinary incontinence was similar between the control group and the tubeless group. Among the three groups, the completely tubeless group had the highest percentage (96.1%) of patients with 0-degree discomfort, whereas the control group had more patients (14.1%) with III-degree discomfort (Supplementary Table 5 and Figure 3).

After PSM, the influences of gender, age, and surgical type and duration were balanced. And the incidence rates of urinary irritation, UTI, and high degree discomfort were still significantly higher in the control group (*p* < 0.001; *p* = 0.009; *p* < 0.001, respectively), while differences did not exist between the control group and the tubeless group in terms of non-automatic micturition and urinary incontinence. Besides, comparing with the partially tubeless group, patients in the completely tubeless group had less UTI and lower degree of discomfort (*p* = 0.019; *p* = 0.002, respectively) (Table 3).

**Table 3:** Operative parameters and postoperative recovery of our study population after PSM.

**Characteristic**	**Full cohort (*n* = 130)**			**Tubeless group (*n* = 54)**		
	**Control group (*n* = 65)**	**Tubeless group (*n* = 65)**	***P***	**Partially tubeless group (*n* = 27)**	**Completely tubeless group (*n* = 27)**	***P***
**Automatic micturition time (h), mean ± SD**			<0.001			0.385
	3.45 ± 3.47	7.25 ± 3.87		6.19 ± 4.15	6.80 ± 3.55	
**Urinary irritation, *n* (%)**			<0.001			0.260
Yes	47 (72.3)	27 (41.5)		12 (44.4)	8 (29.6)	
No	18 (27.7)	38 (58.5)		15 (55.6)	19 (70.4)	
**Urination retention status, *n* (%)**			1.000			0.735
Self-urination	52 (80.0)	52 (80.0)		22 (81.5)	21 (77.8)	
Non-automatic micturition	13 (20.0)	13 (20.0)		5 (18.5)	6 (22.2)	
**Urinary tract infection, *n* (%)**			0.009			0.019
Yes	19 (29.2)	7 (10.8)		5 (18.5)	0 (0.0)	
No	46 (70.8)	58 (89.2)		22 (81.5)	27 (100.0)	
**Urinary incontinence, *n* (%)**			1.000			1.000
Never	63 (96.9)	62 (95.4)		26 (96.3)	25 (92.6)	
Occasionally	2 (3.1)	2 (3.1)		1 (3.7)	2 (7.4)	
Frequently	0 (0.0)	1 (1/5)		0 (0.0)	0 (0.0)	
**Subjective discomfort, *n* (%)**			<0.001			0.002
0 degree	19 (29.2)	54 (83.1)		16 (59.3)	26 (96.3)	
I degree	21 (32.3)	10 (15.4)		10 (37.0)	1 (3.7)	
II degree	14 (21.5)	1 (1.5)		1 (3.7)	0 (0.0)	
III degree	11 (16.9)	0 (0.0)		0 (0.0)	0 (0.0)	
**Time to get out of bed after surgery (h), mean ± SD**			0.035			0.329
	23.08 ± 7.65	19.88 ± 8.74		19.85 ± 8.20	19.37 ± 8.89	
**Duration of postoperative hospital stay (day), mean ± SD**			<0.001			0.172
	5.56 ± 2.19	4.40 ± 1.45		4.92 ± 2.76	4.11 ± 1.28	

*PSM, propensity score–matched; SD, standard deviation*.

### Operative Parameters and Postoperative Recovery

**Figure 4:**
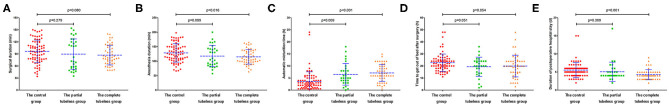
Operation and anesthesia duration and postoperative recovery among groups. **(A)** Operation duration for the three groups; **(B)** anesthesia duration for the three groups; **(C)** automatic micturition time for the three groups; **(D)** time to get out of bed after surgery for the three groups; **(E)** duration of postoperative hospital stay for the three groups.

In the control group, the mean anesthesia and surgical duration was longer than that in the tubeless group (128.42 vs. 115.85 min, *p* = 0.011; 96.28 vs. 87.93 min, *p* = 0.072, respectively). Conversely, there was no difference within the tubeless group. The time to get out of bed after surgery and hospital LOS in the tubeless group were significantly shorter than those in the control group (19.73 vs. 22.72 h, *p* = 0.019; 4.59 vs. 5.53 days, *p* = 0.006, respectively). Additionally, duration of postoperative hospital stay was even shorter in the completely tubeless group than in the partially tubeless group (Supplementary Table 5 and Figure 4).

In order to decrease the influence of surgical type, surgical duration, and anesthesia duration on the postoperative recovery, these factors were balanced using PSM. The advantages of the tubeless protocol in the time to get out of bed and hospital LOS were still significant (*p* = 0.035; *p* < 0.001, respectively) (Table 3).

### Regression Analysis for Predictive Factors of Urinary Complications

Totally, 58 patients suffered urinary complications, including 45 (57.7%) in the control groups, 7 (23.3%) in the partially tubeless group, and 6 (11.8%) in the completely tubeless group (*p* < 0.001). The factors that were significantly associated with the occurrence of urinary complications were age, volume of intraoperative liquid, and catheterization protocol. Multivariable regression analysis showed that only the partially tubeless and the completely tubeless protocol was an independent predictor of the urinary complications [odds ratio (OR) = 0.223, 95% confidence interval (CI) (0.085–0.586), *p* = 0.002; OR = 0.117, 95% CI (0.043–0.319), *p* < 0.001; respectively] (Table 4).

**Table 4:** Regression analysis for predictive factors of urinary complications.

**Variable**	**Univariable analysis**			**Multivariable analysis**		
	**OR**	**95 CI**	***P***	**OR**	**95 CI**	***P***
**Age**						
≤60 years	Reference			Reference		
>60 years	2.391	1.106–5.169	0.027	1.740	0.742–4.081	0.203
**Gender**						
Male	Reference					
Female	0.597	0.308–1.160	0.128			
**Side of operation**						
Left	Reference					
Right	1.061	0.553–2.034	0.859			
**Hypertension**						
No	Reference					
Yes	1.079	0.470–2.478	0.857			
**Heart disease**						
No	Reference					
Yes	0.529	0.028–10.035	0.672			
**Surgical type, *n***						
Wedge resection	Reference					
Segmentectomy	1.168	0.310–4.397	0.819			
Lobectomy	1.348	0.687–2.645	0.385			
**Volume of intraoperative liquid**						
≤1,100 mL	Reference			Reference		
>1,100 mL	2.094	1.061–4.131	0.033	1.296	0.596–2.819	0.513
**Intraoperative blood loss**						
≤75 mL	Reference					
>75 Ml	0.557	0.277–1.122	0.102			
**Diameter of tumor**						
≤2 cm	Reference					
>2 cm	1.766	0.746–4.181	0.196			
**Lymph nodes dissection>10**						
No	Reference					
Yes	0.907	0.236–3.485	0.887			
**Catheterization protocol**						
Conventional	Reference			Reference		
Partially tubeless	0.223	0.086–0.582	0.002	0.223	0.085–0.586	0.002
Completely tubeless	0.098	0.037–0.256	<0.001	0.117	0.043–0.319	<0.001

*CI, confidence interval; OR, odds ratio*.

### Nomogram for Predicting the Risk of Non-automatic Micturition

**Figure 5:**
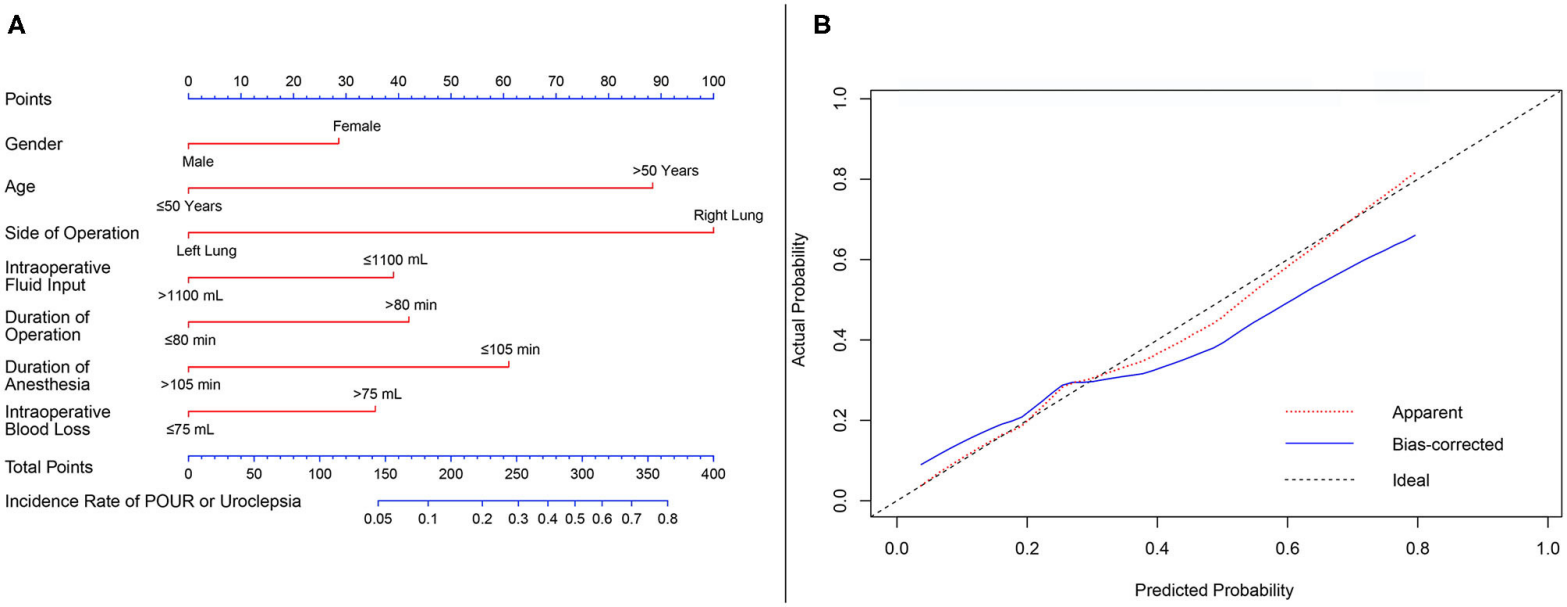
Nomogram predicting the risk of non-automatic micturition in the tubeless group. **(A)** Nomogram predicting the probability of non-automatic micturition; **(B)** calibration curves for the nomogram. min, minutes; mL, milliliter.

As POUR is the most common concern for patients with tubeless catheterization, we constructed a nomogram to predict the incidence of non-automatic micturition in the tubeless group (Figure 5A). Gender, age, side of operation, intraoperative fluid input, duration of operation, duration of anesthesia, and intraoperative blood loss were included in the model. The predictive value of the nomogram was internally validated by a calibration curve that showed optimal agreement between the predicted and actual probabilities (Figure 5B).

## Discussion

The results of this randomized controlled trial indicated that compared with traditional catheterization, tubeless protocol was associated with lower postoperative urinary levels of WBC and RBC, fewer urinary complications, more comfort, earlier mobilization and shorter hospital LOS without increasing the incidence of POUR and urinary incontinence. Furthermore, the advantages of the tubeless procedure were more significant in the completely tubeless group, and it can completely avoid iatrogenic injury of the urethra. The benefits of performing the tubeless protocol still existed even after a 1:1 PSM was performed to balance the influence of gender, age, surgical type, surgical duration, anesthesia duration, and tumor size. Tubeless protocol was considered to be an independent predictor of the occurrence of urinary complications. Additionally, we constructed a nomogram to predict the risk for patients with tubeless catheterization for the first time. This approach can identify high-risk patients among those who underwent the tubeless protocol and used to adopt preventive measures.

ERAS is an evidence-based care improvement process for surgical patients (12). To further reduce the invasiveness of the procedure and accelerate recovery, non-intubated procedures, which use intravenous anesthesia with spontaneous ventilation without the placement of any tube, have been adopted in selected patients to relieve their postoperative pain and facilitate recovery (6). With the development of anesthesia control, non-intubated intravenous anesthesia and early removal of chest tubes have been increasingly employed in pulmonary resection. However, although urinary catheter management is an essential part of ERAS, the clinical advantages of not placing a urinary catheter and the optimal protocol of urinary catheterization have not yet been defined. Thus, our study aimed to provide high-level evidence for the recommendation of urinary catheterization in patients who undergo VATS lung surgery.

In most health care institutions, placement of a urinary catheter until postoperative day 2 is considered standard procedure for pulmonary resection patients to monitor urine output and prevent the incidence of POUR (13). However, the clinical effect of monitoring urine output for uncomplicated pulmonary resection has been questioned. Sachin's study demonstrated that intraoperative urine output was not an independent predictor of acute renal failure for non-cardiac surgery patients whose previous renal function was normal (14). Similarly, in VATS lung resection patients, a study found that intraoperative urinary output and postoperative renal function were not affected by the administration of fluids (15). Thus, except in patients with previously abnormal renal function or for whom fluid management is crucial, such as pneumonectomy, placing a urinary catheter to monitor perioperative urine output is not recommended (11).

POUR was defined as the inability to void in the presence of a full bladder. The development of POUR in VATS lung resection is mainly influenced by the type of anesthesia. Thoracic epidural analgesia (TEA) is associated with a high incidence of POUR (13). Prevention of POUR is also considered a potential advantage of indwelling catheterization. Mark's study reported that early removal of urinary catheters was associated with a higher rate of POUR, and a greater number of patients required reinsertion of urinary catheters when an epidural catheter was still in place after a thoracic operation (16). Compared to TEA, PB was believed to reduce the risks of developing POUR, while the effect of analgesia is similar (17). In our study, all patients underwent intravenous anesthesia with PB, and there was no difference in the incidence of POUR or urinary incontinence among patients with traditional catheterization or tubeless protocol. Moreover, as POUR was the most common concern for medical staff to perform the tubeless protocol, we also constructed a nomogram to predict the incidence rate of non-automatic micturition. This nomogram not only considers the preoperative characteristics but also takes operative factors into account. For patients with long duration of anesthesia and at high risk of POUR, the urine catheter should not be removed for patients who underwent the partially tubeless protocol, or a urine catheterization should be performed to prevent POUR for those who planned to choose the completely tubeless protocol. Other preventive measures such as more frequent bedside ultrasound scans and induction therapy should also be considered.

UTI has long been considered the most common health care–associated infection, with the vast majority of these infections occurring after placement of often unnecessary urinary catheters (18). The risk of UTI increases with prolonging duration of urinary catheter placement, and a previous study reported that 24% of patients will develop symptomatic UTI, and bacteremia will develop in 3.6% after 2–10 days of catheterization (19). Thus, avoiding unnecessary urinary catheter placement is the most important strategy in the prevention of UTI. However, the urinary catheter use has been reported to be inappropriate for 21% of patients, and continued catheterization was judged to be appropriate for almost one-half of catheter-days (20). Current guidelines limit the acceptable indications for the use of indwelling urinary catheters, including significant POUR, refractory urinary incontinence, and accurate urine output monitoring (10). In our study, compared with traditional catheterization, tubeless catheterization was associated with lower incidence rate of UTI, pyuria, and hematuria. More importantly, patients with completely tubeless catheterization had an even lower UTI rate than those with partially tubeless catheterization, which indicated that traditional catheterization is unnecessary and carries a higher risk of UTI for patients met our criteria, and not placing of a urinary catheter can further decrease the incidence of UTI. Moreover, once UTI was diagnosed, antibiotics should be used to completely cure the infection, which also prolonged the length of hospital stay.

Early removal of the urinary catheter could also be crucial because it can motivate early mobilization, which is essential for ERAS. Proceeding with early discontinuation of urinary catheters could motivate early mobilization, which lead to prevention of thrombosis and early hospital discharge (21). In our study, patients with tubeless catheterization had a shorter postoperative in-bed duration and shorter hospital LOS than patients with traditional catheterization. The advantages were more significant when no urinary catheter was inserted.

The core emphasis of ERAS is to minimize stress (12). Adequate discomfort relief can attenuate neurohormonal reflexes, minimize the risk of organ dysfunction, and reduce complications (22). Our data are consistent with this concept and demonstrate that the tubeless protocol was associated with more comfort and better pain relief, while not placing of a urinary catheter showed the best outcome without the incidence of degree II–III discomfort.

Previous studies reported that the incidence of iatrogenic catheter-related urinary injury is 0.3–3.0%, and the most common ways of injury by urinary catheterization were inadvertent balloon inflation in the urinary tract during insertion (23, 24). The iatrogenic injury of the urethra and related complications were associated with significant cost and longer hospital LOS (25). Although some studies were conducted to design a safer urinary catheter system to prevent catheterization-related urinary injuries (26, 27), iatrogenic urinary injuries continued to occur. However, for patients without catheterization in our study, the risk of iatrogenic injury of the urethra could be avoided.

Previous studies on urinary catheterization after lung resection have mainly focused on the outcome of the early removal of urinary catheter and the association between duration of catheter placement and the incidence of UTIs or POUR. However, a consensus on the timing of removal still cannot be made, and no validated evidence has identified the feasibility of tubeless catheterization (11). To the best of our knowledge, this is the first study that proposed specific criteria for unnecessary catheterization and conducted a prospective randomized trial to compare the outcomes of three different types of catheterizations. Our results showed that for selected patients under intravenous anesthesia with PB, performing of the tubeless protocol was associated with earlier postoperative mobilization, less discomfort, lower risks of UTI, and shorter hospital LOS without increasing the incidence of POUR. Compared with traditional catheterization, tubeless catheterization was considered to be an independent protective factor for urinary complications. Most importantly, the completely tubeless catheterization had the lowest incidence rate of UTI and discomfort with the shortest hospital LOS, which provided evidence for the feasibility and advantages of avoiding urinary catheter placement in selected patients. The specific criteria for selecting patients were also proven to be safe and practical. In case of POUR, we further constructed a nomogram to predictive the risk of non-automatic micturition. However, for patients who have a history of urinary system disease and underwent TEA and in whom fluid balance is crucial, traditional catheterization should still be performed.

There are several limitations to the present study. As we restricted inclusion to patients, the findings might not be generalizable for all patients who underwent VATS lung surgery. However, from our clinical experience, we excluded no more than a quarter of the high-risk population, which implies that the exclusion proportion is small. Additionally, given the intrinsic characteristics of our study, it was impossible to apply double blinding.

## Conclusion

The tubeless protocol led to less complications, earlier mobilization, more comfort, and shorter hospital LOS without increasing the rate of POUR. The completely tubeless protocol was more advantageous. This new protocol is feasible and should be recommended for patients who meet our criteria. The utility of our nomogram can assist clinicians in avoiding risks in performing the tubeless protocol.

## Data Availability Statement

The raw data supporting the conclusions of this article will be made available by the authors. We may balance the potential benefits and risks for each request and then provide the data that could be shared.

## Ethics Statement

This study was approved by the Medical Ethics Committee of the First Affiliated Hospital, School of Medicine, Zhejiang University, the reference number is 2016-249. Every individual participant had signed an informed consent form for participating in the study.

## Author Contributions

WW: conceptualization, formal analysis, data curation, software, visualization, writing–original draft, and writing–review and editing. JH: conceptualization, data curation, funding acquisition, project administration, resources, supervision, validation, and writing–review and editing. WL: investigation and visualization. JX: methodology and visualization. LP: investigation and software. PX: conceptualization, investigation, methodology, and writing–original draft.

## Conflict of Interest

The authors declare that the research was conducted in the absence of any commercial or financial relationships that could be construed as a potential conflict of interest.

## Abbreviations

CI: confidence interval
ERAS: Enhanced Recovery After Surgery
LOS: length of stay
OR: odds ratio
PB: paravertebral blockade
POUR: postoperative urinary retention
RBC: red blood cell
TCI: target control infusion
TEA: thoracic epidural analgesia
UTI: urinary tract infection
VATS: video-assisted thoracoscopic surgery
WBC: white blood cell.
