# Supplementary material for: A Novel Tubeless Urinary Catheter Protocol Enhanced Recovery After Minimally Invasive Lung Surgery
Source: Front Surg. 2020 Nov 9;7:584578. doi: 10.3389/fsurg.2020.584578 (PMC7693547; doi:10.3389/fsurg.2020.584578)
Supplement: Supplementary file 1 [file Table_1.DOCX]

**Supplement Table 1.** **The eligibility and exclusion criteria.**

| The eligibility criteria | |
| --- | --- |
| 1 | patients who had no contraindications to lung surgery and signed informed consent; |
| 2 | patients with American Anesthesiologists Association (ASA) scores I~II; |
| 3 | patients who underwent elective VATS lung resection that lasted less than 150 minutes; |
| 4 | patients older than 18 and younger than 70 years of age; |
| 5 | patients with normal renal and bladder function preoperatively; |
| 6 | patients who had systematic urination exercise prior to operation; |
| 7 | patients without a known preoperative urinary tract infection. |
| The exclusion criteria | |
| 1 | patients with severe urinary system diseases (including urinary calculi, trauma, infection, renal failure, acute kidney injury, proteinuria, kidney transplantation and tumors) or administration of potentially nephrotoxic substances before surgery; |
| 2 | patients with severe systemic diseases (such as congestive heart failure, hepatic failure) or body mass indexes >35 kg/m^2^; |
| 3 | patients with a history of an indwelling urinary catheter or urostomy or who were being intermittently catheterized preoperatively; |
| 4 | patients with history or symptoms of benign prostatic hyperplasia, chronic prostatitis and urinary retention; |
| 5 | patients with a history of lower urinary tract surgery; |
| 6 | patients who required a urologist to insert the urinary catheter at the time of the operation; |
| 7 | patients with intraoperative hemorrhage and the need for blood transfusion; |
| 8 | patients with other risk factors for developing postoperative acute renal failure (such as peripheral vascular occlusive disease, chronic obstructive pulmonary disease necessitating chronic bronchodilator therapy, use of a vasopressor infusion, diuretic administration); |
| 9 | patients who needed strict urine output monitoring; |
| 10 | patients with severe nonurinary system-related postoperative complications (such as hemodynamic instability or acute respiratory distress syndrome or venous thromboembolism or who died in the hospital); |
| 11 | patients who themselves or whose family members had refused removal of the catheter immediately after surgery or who dropped out from the study. |
