# Supplementary material for: A Novel Tubeless Urinary Catheter Protocol Enhanced Recovery After Minimally Invasive Lung Surgery
Source: Front Surg. 2020 Nov 9;7:584578. doi: 10.3389/fsurg.2020.584578 (PMC7693547; doi:10.3389/fsurg.2020.584578)
Supplement: Supplementary file 2 [file Table_2.DOCX]

**Supplement Table 2. Questionnaire of postoperative parameters.**

| Q1 | Objective discomfort? |
| --- | --- |
| A | 0 degree, no discomfort; |
| B | I degree, mild discomfort (VAS score 1~3), reflected only after being asked by medical staff; |
| C | II degree, moderate discomfort (VAS score 4~7), a complaint by patients who revealed an uncomfortable sensation in the urethra or urethral foreign body sensation without clear agitation; |
| D | III degree, severe discomfort (VAS score 8~10), spontaneous agitation, continuous complaints and urgent to remove the catheter associated with an extremely uncomfortable sensation in the urethra. |
| Q2 | Postoperative urine retention state? |
| A | Voluntarily urinate; |
| B | Induced uresis, patients can’t urinate automatically, but can urinate after body position changes, hot compress on bladder area or vulva wash by warm water; |
| C | Urine retention, inability to voluntarily urinate or a residual urine volume greater than 600ml, diagnosed by bedside ultrasound; |
| Q3 | Symptoms of urinary track infection? |
| A | Urinary irritation; |
| B | New onset or worsening of fever, rigors, altered mental status, malaise or lethargy with no other identified cause; |
| C | Flank pain; |
| D | Costovertebral angle tenderness; |
| E | Acute hematuria; |
| F | Pelvic discomfort; |
| G | Dysuria, urgent or frequent urination, or suprapubic pain or tenderness after catheters removed. |
| Q4 | Postoperative day one activity state? |
| A | Lie on the bed; |
| B | Move on the bed; |
| C | Move around the bed; |
| D | Walk in the room. |
| Q5 | Incidence of uroclepsia? |
| A | Never; |
| B | Occasionally; |
| C | Sometimes; |
| D | Often. |
| *VSA*, visual analogue score. | |
