# Supplementary material for: A Novel Tubeless Urinary Catheter Protocol Enhanced Recovery After Minimally Invasive Lung Surgery
Source: Front Surg. 2020 Nov 9;7:584578. doi: 10.3389/fsurg.2020.584578 (PMC7693547; doi:10.3389/fsurg.2020.584578)
Supplement: Supplementary file 3 [file Table_3.DOCX]

**Supplement Table 3. Baseline characteristics of the study population after PSM.**

| **Characteristic** | **Full Cohort (n=130)** | | | **Tubeless Group (n=54)** | | |
| --- | --- | --- | --- | --- | --- | --- |
|  | **Control Group**  **(n=65)** | **Tubeless Group**  **(n=65)** | ***P value*** | **Partially tubeless Group**  **(n=27)** | **Completely tubeless Group**  **(n=27)** | ***P value*** |
| **Age, n (%)** |  |  | *0.523* |  |  | *0.100* |
| ≤60 years | 49 (75.4%) | 53 (81.5%) |  | 6 (22.2%) | 1 (3.7%) |  |
| >60 years | 16 (24.6%) | 12 (18.5%) |  | 21 (77.8%) | 26 (96.3%) |  |
| **Gender, n (%)** |  |  | *0.587* |  |  | *0.551* |
| Male | 26 (40.0%) | 23 (35.4%) |  | 7 (25.9%) | 9 (33.3%) |  |
| Female | 39 (60.0%) | 42 (64.6%) |  | 20 (74.1%) | 18 (66.7%) |  |
| **Side of operation, n (%)** |  |  | *0.724* |  |  | *0.783* |
| Left | 30 (46.2%) | 28 (43.1%) |  | 12 (44.4%) | 11 (40.7%) |  |
| Right | 35 (53.8%) | 37 (56.9%) |  | 15 (55.6%) | 16 (59.3%) |  |
| **Diabetes, n (%)** |  |  | *1.000* |  |  | *1.000* |
| Yes | 2 (3.1%) | 2 (3.1%) |  | 0 (0.0%) | 1 (3.7%) |  |
| No | 63 (96.9%) | 63 (96.9%) |  | 27 (100.0%) | 26 (96.3%) |  |
| **Hypertension, n (%)** |  |  | *0.517* |  |  | *0.467* |
| Yes | 12 (18.5%) | 15 (23.1%) |  | 6 (22.2%) | 3 (11.1%) |  |
| No | 53 (81.5%) | 50 (76.9%) |  | 21 (77.8%) | 24 (88.9%) |  |
| **Surgical type, n (%)** |  |  | *0.358* |  |  | *0.574* |
| Lobectomy | 32 (49.2%) | 31 (47.7%) |  | 12 (44.4%) | 12 (44.4%) |  |
| Segmentectomy | 7 (10.8%) | 3 (4.6%) |  | 0 (0.0%) | 2 (7.4%) |  |
| Wedge resection | 26 (40.0%) | 31 (47.7%) |  | 15 (55.6%) | 13 (48.2%) |  |
| **Intraoperative blood loss, (milliliter), mean±SD** |  |  | *0.212* |  |  | *0.591* |
|  | 71.18±29.94 | 66.12±21.74 |  | 70.44±22.68 | 67.26±21.43 |  |
| **Surgical duration, (minute), mean ± SD** |  |  | *0.441* |  |  | *0.953* |
|  | 92.88±28.60 | 89.74±30.91 |  | 84.00±35.61 | 83.52±22.41 |  |
| **Anesthesia duration, (minute), mean ± SD** |  |  | *0.399* |  |  | *0.925* |
|  | 122.46±29.98 | 118.68±30.25 |  | 111.67±32.81 | 112.41±24.05 |  |
| **Pathological diagnosis, n (%)** |  |  | *0.119* |  |  | *0.352* |
| Malignance | 56 (86.2%) | 49 (75.4%) |  | 22 (81.5%) | 18 (66.7%) |  |
| Benign disease | 9 (13.8%) | 16 (24.6%) |  | 5 (18.5%) | 9 (33.3%) |  |
| **Diameter of tumor, (centimeter), mean±SD** |  |  | *0.622* |  |  | *0.366* |
|  | 1.44±1.02 | 1.33±0.88 |  | 1.16±0.85 | 1.23±0.63 |  |
| **Lymph node dissection, n (%)** |  |  | *0.303* |  |  | *0.544* |
| Yes | 52 (80.0%) | 47 (72.3%) |  | 21 (77.8%) | 18 (66.7%) |  |
| No | 13 (20.0%) | 18 (27.7%) |  | 6 (22.2%) | 9 (33.3%) |  |
| *PSM,* propensity score-matched*; SD*, standard deviation. | | | | | | |
