# Supplementary material for: A Novel Tubeless Urinary Catheter Protocol Enhanced Recovery After Minimally Invasive Lung Surgery
Source: Front Surg. 2020 Nov 9;7:584578. doi: 10.3389/fsurg.2020.584578 (PMC7693547; doi:10.3389/fsurg.2020.584578)
Supplement: Supplementary file 4 [file Table_4.DOCX]

**Supplement Table 4. Urine** **laboratory examination of our study population.**

| **Characteristic** | **Full Cohort (n=159)** | | | **Tubeless Group (n=81)** | | |  |
| --- | --- | --- | --- | --- | --- | --- | --- |
|  | **Control Group**  **(n=78)** | **Tubeless Group**  **(n=81)** | ***P value*** | **Partially tubeless Group (n=30)** | **Completely tubeless Group (n=51)** | ***P value*** | |
| **Postoperative urine white blood cell, median (min, max)** |  |  | *<0.001* |  |  | *0.131* |  |
|  | 23.9(0.3, 1651.0) | 6.6(0.0, 207.4) |  | 8.1(0.0, 207.4) | 5.5(0.0, 143.3) |  |  |
| **Postoperative pyuria, n (%)** |  |  | *<0.001* |  |  | *0.752* |  |
| Positive | 48(61.5) | 20(24.7) |  | 8(26.7) | 12(23.5) |  |  |
| Negative | 30(38.5) | 61(75.3) |  | 22(73.3) | 39(76.5) |  |  |
| **Postoperative urine red blood cell, median (min, max)** |  |  | *<0.001* |  |  | *0.454* |  |
|  | 22.8(1.0,1116.8) | 7.9(0.0, 228.7) |  | 9.4(0.0, 68.0) | 7.3(0.0,228.7) |  |  |
| **Postoperative haematuria, n (%)** |  |  | *<0.001* |  |  | *0.792* |  |
| Positive | 43(55.1) | 15(18.5) |  | 6(20.0) | 9(17.6) |  |  |
| Negative | 35(44.9) | 66(81.5) |  | 24(80.0) | 42(82.4) |  |  |
| *Max,* maximum*; Min,* minimum*.* | | | | | | |  |
