# Supplementary material for: A Novel Tubeless Urinary Catheter Protocol Enhanced Recovery After Minimally Invasive Lung Surgery
Source: Front Surg. 2020 Nov 9;7:584578. doi: 10.3389/fsurg.2020.584578 (PMC7693547; doi:10.3389/fsurg.2020.584578)
Supplement: Supplementary file 5 [file Table_5.DOCX]

**Supplement Table 5. Operative parameters and postoperative recovery of our study population.**

| **Characteristic** | **Full Cohort (n=159)** | | | **Tubeless Group (n=81)** | | |
| --- | --- | --- | --- | --- | --- | --- |
|  | **Control Group**  **(n=78)** | **Tubeless Group**  **(n=81)** | ***P value*** | **Partially tubeless Group (n=30)** | **Completely tubeless Group (n=51)** | ***P value*** |
| **Surgical duration, (minute), mean ± SD** |  |  | *0.060* |  |  | *0.752* |
|  | 96.28±28.33 | 87.93±29.87 |  | 89.47±37.73 | 87.02±24.49 |  |
| **Anesthesia duration, (minute), mean ± SD** |  |  | *0.008* |  |  | *0.735* |
|  | 128.42±31.65 | 115.85±29.62 |  | 117.47±36.51 | 114.90±25.06 |  |
| **Automatic micturition time, (hour), mean ± SD** |  |  | *<0.001* |  |  | *0.517* |
|  | 3.62±3.97 | 6.90±3.86 |  | 6.54±4.49 | 7.12±3.47 |  |
| **Urinary irritation, n (%)** |  |  | *<0.001* |  |  | *0.353* |
| Yes | 58(74.4) | 32(39.5) |  | 14(46.7) | 18(35.3) |  |
| No | 20(25.6) | 49(60.5) |  | 16(53.3) | 33(64.7) |  |
| **Urination retention status, n (%)** |  |  | *0.771* |  |  | *0.966* |
| Self-urination | 64(82.1) | 65(80.2) |  | 24(80.0) | 41(80.4) |  |
| non-automatic micturition | 14(17.9) | 16(19.8) |  | 6(20) | 10(19.6) |  |
| **Urinary tract infection, n (%)** |  |  | *0.001* |  |  | *0.049* |
| Yes | 22(28.2) | 7(8.6) |  | 5(16.7) | 2(3.9) |  |
| No | 56(71.8) | 74(91.4) |  | 25(83.3) | 49(96.1) |  |
| **Urinary incontinence, n (%)** |  |  | *0.616* |  |  | *0.645* |
| Never | 74(94.9) | 76(93.8) |  | 29(96.7) | 47(92.1) |  |
| Occasionally | 4(5.1) | 4(4.9) |  | 1(3.3) | 3(5.9) |  |
| Frequently | 0(0.0) | 1(1.3) |  | 0(0.0) | 1(2.0) |  |
| **Subjective discomfort, n (%)** |  |  | *<0.001* |  |  | *<0.001* |
| 0 degree | 24 (30.8) | 66 (81.5) |  | 17 (56.7) | 49 (96.1) |  |
| I degree | 26 (33.3) | 14 (17.3) |  | 12 (40.0) | 2 (3.9) |  |
| II degree | 17 (21.8) | 1 (1.2) |  | 1 (3.3) | 0 (0.0) |  |
| III degree | 11 (14.1) | 0 (0.0) |  | 0 (0.0) | 0 (0.0) |  |
| **Time to get out of bed after surgery, (hour), mean ± SD** |  |  | *0.035* |  |  | *0.773* |
|  | 22.72±7.20 | 19.73±8.56 |  | 19.37±7.94 | 19.94±8.98 |  |
| **Duration of postoperative hospital stay, (day), mean ± SD** |  |  | *<0.001* |  |  | *0.106* |
|  | 5.53±2.12 | 4.59±2.01 |  | 5.07±2.70 | 4.31±1.42 |  |
| *SD,* standard deviation. | | | | | | |
