# Supplementary figures and images for: A Novel Tubeless Urinary Catheter Protocol Enhanced Recovery After Minimally Invasive Lung Surgery
Source: Front Surg. 2020 Nov 9;7:584578. doi: 10.3389/fsurg.2020.584578 (PMC7693547; doi:10.3389/fsurg.2020.584578)

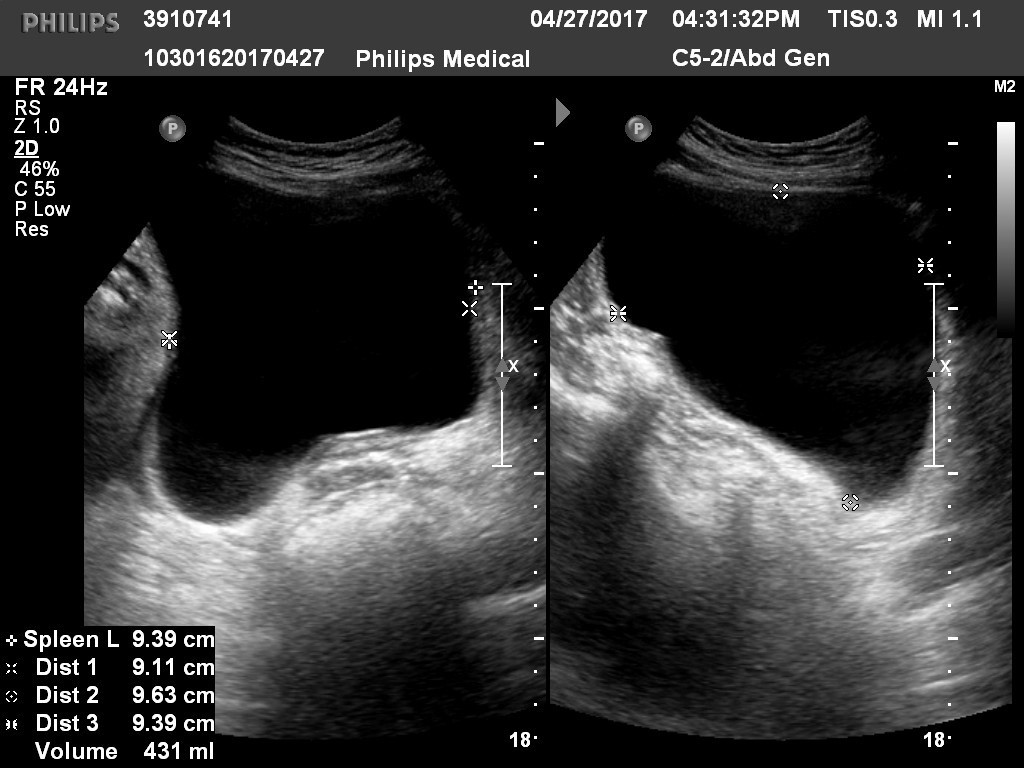

Supplement: Supplementary file 6 [file Image_1.TIF]
